# An Overview of the Neurotrophic and Neuroprotective Properties of the Psychoactive Drug Lithium as an Autophagy Modulator in Neurodegenerative Conditions

**DOI:** 10.7759/cureus.44051

**Published:** 2023-08-24

**Authors:** Ajay Singh, Sanjiya Arora, Manisha Chavan, Samen Shahbaz, Hafsa Jabeen

**Affiliations:** 1 Internal Medicine, Sri Ram Murti Smarak Institute of Medical Sciences, Bareilly, IND; 2 Health Department, Sub District Hospital (SDH) cum Civil Hospital, Fatehabad, Fatehabad, IND; 3 Internal Medicine, Kakatiya Medical College, Rangam Peta, Warangal, IND; 4 Internal Medicine, Faisalabad Medical University, Faisalabad, PAK; 5 Internal Medicine, Dow University of Health Sciences, Nanakwara, PAK

**Keywords:** amyotrophic lateral sclerosis, parkinson’s disease, alzheimer’s disease, huntingtin’s disease, neuroprotective, bipolar disorder, lithium, psychoactive drugs, autophagy

## Abstract

For both short-term and long-term treatment of bipolar disorder, lithium is a prototypical mood stabilizer. Lithium's neuroprotective properties were revealed by cumulative translational research, which opened the door to reforming the chemical as a treatment for neurodegenerative illnesses. The control of homeostatic systems such as oxidative stress, autophagy, apoptosis, mitochondrial function, and inflammation underlies lithium's neuroprotective characteristics. The fact that lithium inhibits the enzymes inositol monophosphatase (IMPase) and glycogen synthase kinase (GSK)-3 may be the cause of the various intracellular reactions. In this article, we review lithium's neurobiological properties, as demonstrated by its neurotrophic and neuroprotective capabilities, as well as translational studies in cells in culture and in animal models of Alzheimer's disease (AD), Parkinson's disease (PD), Huntington's disease (HD), Prion disease, amyotrophic lateral sclerosis (ALS), ischemic stroke, and neuronal ceroid lipofuscinosis (NCL), discussing the justification for the drug's use in the treatment of these neurodegenerative disorders.

## Introduction and background

**Figure 1:**
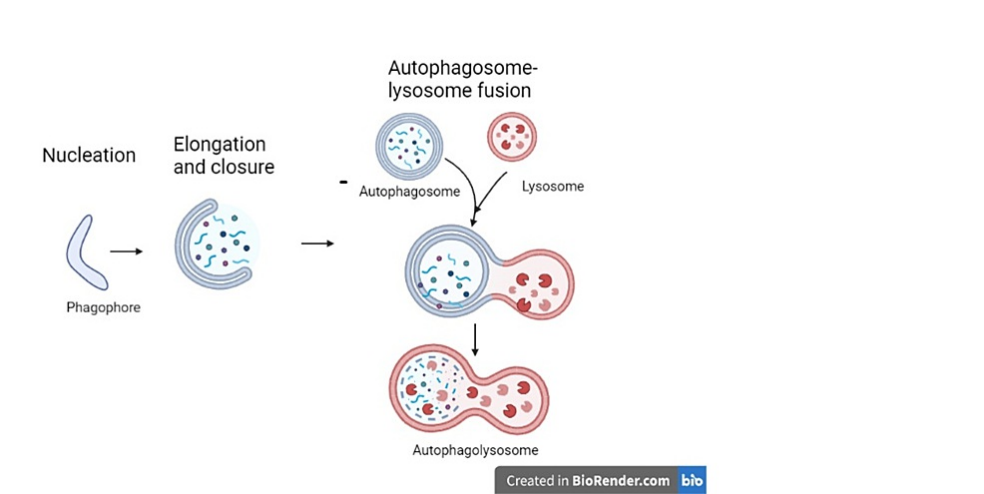
The macroautophagy process Macroautophagy takes place in five main steps: (1) Induction occurs after a metabolic or therapeutic stress, and is mediated by a complex containing the ULK1 protein. (2) During the nucleation step, the formation of the phagophore (or isolation membrane) is initiated. This action is mainly triggered by a protein complex containing VPS34, a PtdIns3K of class III. (3) Elongation of the phagophore involves two «ubiquitin like» conjugation systems: the Atg12-Atg15 and the Atg8-PE, which are required for autophagosome formation. (4) Once formed, the autophagosome containing a cytosolic cargo will fuse with the lysosome, which triggers (5) the degradation of its content and the release of primary components in the cytosol for recycling. The lysosome can then be regenerated so that the process can start again.

The ubiquitin-proteasome system and the autophagy-lysosome pathway are the two main mechanisms used by eukaryotic cells to degrade proteins. Initiation, elongation, maturation, and fusion are the four phases that make up the 'self-eating' process of autophagy (here referred to as macroautophagy) (Figure 1) [1]. The development of a cup-shaped structure in the cytoplasm known as the phagophore starts this process. More proteins are gathered by the phagophore, allowing the membrane to lengthen and form the autophagosome, a double membrane-bound structure. Together with the cellular components to be broken down, a portion of the cytoplasm is contained in the autophagosome. To increase the likelihood that autophagosomes and lysosomes will fuse to produce autophagolysosomes, autophagosomes are subsequently transported by microtubules to the perinuclear region of the cell. The protein and organelle contents of the autophagosome are broken down by acidic lysosomal hydrolases after fusion with lysosomes and recycled. Proton pumps called vacuolar H+ ATPases, which are found inside lysosomal membranes, allow the autolysosome contents to be acidified. The activation of lysosomal enzymes like cathepsins or other acid hydrolases, which are in charge of proteolyzing the components in the autophagolysosome, depends on this acidity [2]. Both health and illness involve the physiological functions of autophagy. When deprived of nutrients, autophagy non-selectively catabolizes cytoplasmic components into building blocks like amino acids. Even in nutrient-rich environments, autophagy also occurs constitutively at low levels and mediates the overall turnover of cytoplasmic components. Constitutive autophagy serves as the cytoplasmic quality-control system and is essential for maintaining homeostasis in a variety of post-mitotic cells, including neurons. Non-selective autophagy may be able to partially accomplish this quality control, but mounting evidence suggests that "selective" autophagy targets particular proteins, organelles, and invasive microorganisms [3].

## Review

Methods

A systematic search was conducted from July 2022 to December 2022 following the guidelines of the Preferred Reporting Items for Systematic Review and Meta-analysis (PRISMA) to identify the relevant studies through PubMed, Google Scholar, and the Cochrane database. Lithium combined with mechanisms using the AND operator and mechanisms OR neurodegenerative conditions were among the search phrases. Studies were limited to those involving adult human subjects, written in the English language, and published over the past 25 years (January 1998 to February 2023). A total of 316 full-text articles were assessed for eligibility. After excluding the review articles, animal studies, opinion papers, case reports, secondary data, letters to editors, and inadequate design, 28 studies were included in the qualitative synthesis.

Regulatory mechanisms and signaling pathways in autophagy

A well-known inhibitor of autophagy, mammalian target rapamycin (mTOR), works by inhibiting the activity of the ULK1 complex. The presence of amino acids and growth factors, as well as the cell's energy and nutrient status, are only a few of the upstream cues that affect mTOR activity. The execution of autophagy depends on a large number of proteins that are encoded by ATg genes and located downstream of mTOR [4]. BH3-only proteins, the inositol 1,4,5-triphosphate receptor (IP3R), the 5-AMP-activated protein kinase (AMPK), the stress-activated enzyme Jun N-terminal kinase 1 (JNK1), Erk 1/2, and calcium are examples of pathways that function independently of mTOR [5-7]. Additionally, autophagy can be initiated pharmacologically by either blocking negative regulators like mTOR with the drug rapamycin [8] or by mTOR-independent inducers such as trehalose [9]. 3-Methyladenine (3-MA), wortmannin, and LY 294002 [10,11], as well as chloroquine and hydroxychloroquine, are pharmacological inhibitors of autophagy.

**Figure 2:**
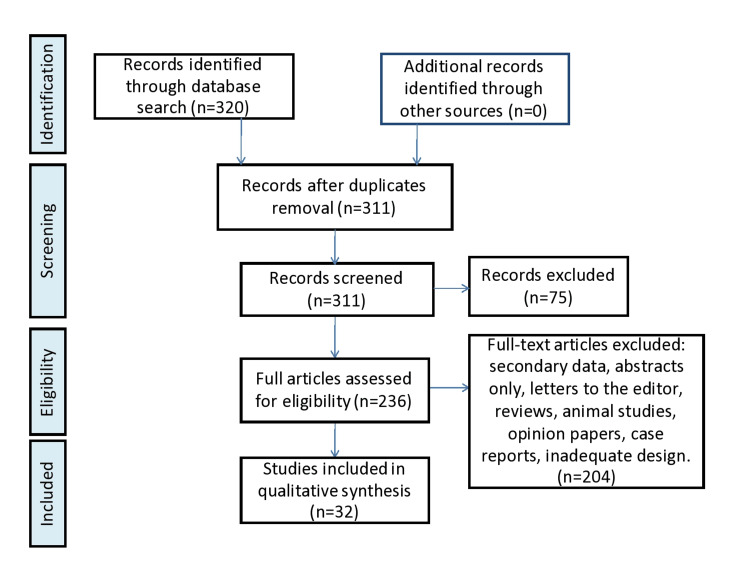
PRISMA flowchart of our review's results

Several enzymes, notably inositol monophosphatase (IMPase), have been suggested as potential targets of lithium activity. Carmichael et al. [12] were the first to describe lithium's ability to induce autophagy, which helps to improve the clearance of proteins that are prone to aggregation, like mutant alpha-synuclein and huntingtin. Lithium's suppression of GSK3β increased mTOR activity, which decreased autophagy [13,14]. The inhibition of IMPase by lithium, on the other hand, caused autophagy to be triggered without the involvement of mTOR [14]. Inositol monophosphate (IP1) must be hydrolyzed into free inositol by IMPase in order for the phosphoinositol signaling pathway to function [13,15]. Lithium impacts this pathway by blocking IMPase, which results in the depletion of free inositol and a subsequent drop in myo-inositol-1,4,5-triphosphate (IP3) levels (Figure 2). Lithium's impact is reversed when inositol or IP3 levels are elevated. Autophagy has been shown to be suppressed by IP3 and the stimulation of its receptor [16]. Lithium, carbamazepine (CBZ), and valproic acid (VPA) are examples of mood-stabilizing medications that use the common mechanism of inositol depletion [6]. CBZ and VPA also improved the clearance of proteins that are prone to aggregation, which is consistent with the role of inositol depletion in autophagy regulation [17]. In a fly model for Huntington's disease (HD), treatment with the mTOR inhibitor rapamycin in conjunction with lithium is more protective than treatment with either drug alone [16]. Both mTOR-dependent (mTOR inhibition by rapamycin) and mTOR-independent (IMPase inhibition by lithium) pathways are enhanced by this combination. With mTOR inhibition plus lithium, the approach demonstrated higher protection against neurodegeneration in an HD fly model than with either pathway alone [14].

**Figure 3:**
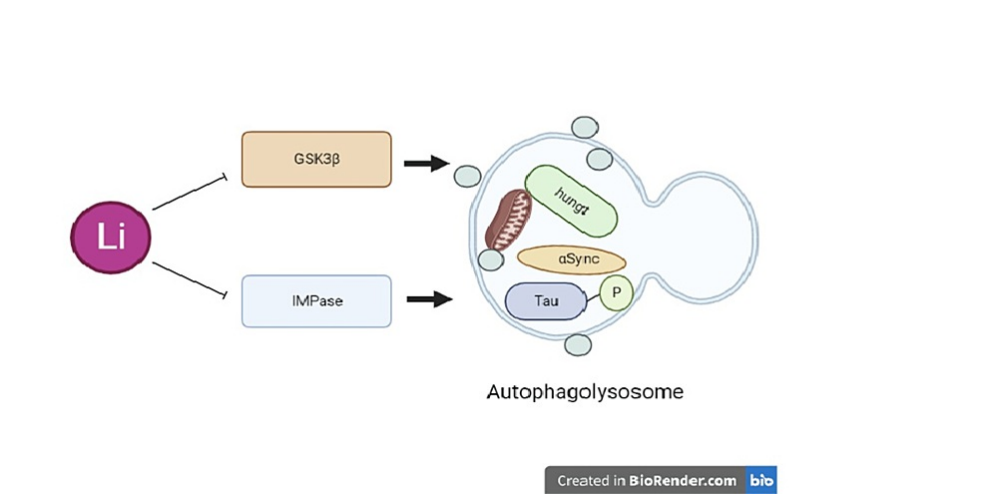
The signaling pathway of lithium as an autophagy enhancer might be associated with the mammalian target of rapamycin (mTOR)-independent pathway, which is involved in myoinositol-1,4,5-trisphosphate (IP3) in Huntington’s disease and Parkinson’s disease. However, the mTOR-dependent pathway might be involved in inhibiting glycogen synthase kinase-3β (GSK3β) in other diseases.

Glycogen synthase kinase-3β (GSK3β) has a wide range of biological functions, including cell death, cell cycle, and carcinogenesis, and is a key regulator of different signal transduction pathways. Lithium is a direct and indirect inhibitor of GSK3β, which probably negatively impacts autophagy [18-19] (Figure 3). Site-specific phosphorylation controls the activity of GSK3. Phosphorylation of the Tyr216 residue increases GSK3β activity, while Ser9 phosphorylation decreases GSK3β activity. The protein kinase ULK1 is necessary for metazoan autophagy and is directly acetylated and stimulated by the activation of the acetyl-transferase TIP60 (HIV-Tat interaction protein, 60 kDa) by GSK3 during serum-deprivation-induced autophagy [20].

Characteristics of neuronal autophagy

A number of diseases can result from improper regulation of the autophagy machinery and/or dysfunction of the lysosomal process, which can disturb cellular homeostasis. Neurodegenerative illnesses are characterized by three primary dysregulations: (1) insufficient autophagy activation; (2) autophagy malfunction brought on by impaired lysosomal function; and (3) autophagic stress driven by pathological autophagy activation. These findings suggest that autophagy normally has a significant neuroprotective function. It has been noted that the autophagic mechanism generally safeguards neurons. Synaptic plasticity, glial cell anti-inflammatory activity, oligodendrocyte formation, and the myelination process all depend on neuronal autophagy [21-22].

As neurons are post-mitotic cells, cell division cannot dilute the aggregate-prone proteins in neurons. As a result, neurons need effective protein quality control systems. Similarly, aberrant protein buildup brought on by altered protein degradation system activity eventually results in neuronal dysfunction, including unregulated transcription and reduced axonal trafficking [23]. A growing body of research suggests that neurological illnesses like Alzheimer's disease (AD), Parkinson's disease (PD), Huntington's disease, amyotrophic lateral sclerosis (ALS), and multiple sclerosis (MS) are associated with autophagy.

Targeting lithium as an autophagy modulator in various neuropsychiatric diseases

Huntington’s Disease

Choreatic movements, psychiatric issues, striatal atrophy with targeted tiny neuron loss, and autosomal dominant inheritance are all traits of HD. For the treatment of this degenerative illness, more modern therapeutic approaches are being developed. One of these categories of drugs now being tested is neuroprotective agents. Lithium assisted in the stabilization of their patients' moods and in preventing chorea progression, according to Danivas et al. [24]. The autosomal dominant gene huntingtin contains extended polyglutamine cytosine-adenine-guanine (CAG) repeat sequences that result in mutant huntingtin protein, which is the etiology of HD [25]. Aggregates of mutant huntingtin are seen inside neurons. Lithium increased the clearance of autophagy substrates, including mutant huntingtin fragments [13]. As previously mentioned, lithium inhibited IMPase, which caused a drop in inositol levels [16].

Lithium treatment decreased the expression of the histone deacetylase 1 protein in HEK293T and Hela cells that had been transfected with mutant huntingtin and aided in the autophagic clearance of mutant huntingtin [26]. The authors conclude that lithium-facilitated downregulation of HDAC1 is independent of GSK3β because GSK3β inhibitors had no effect on the level of HDAC1. Therefore, it is probable that lithium's function to increase autophagy in relation to mutant huntingtin occurs independently of GSK3β. In HD patients, lithium may also be successful in reducing suicidal symptoms [27].

Alzheimer’s Disease

Neuronal and glial autophagy control the pathophysiology of AD. Depletion of autophagy-related proteins in neurons inhibits neuronal autophagy, which in turn leads to the buildup of Aβ species and neurofibrillary tangles and, in the case of AD models, eventual neurodegeneration. The clearance of Aβ species and neurofibrillary tangles in AD models is promoted by the overexpression of autophagy proteins, which lessens AD pathogenesis [28]. Depletion of autophagy-related proteins in microglia inhibits Aβ species neuronal autophagy and leads to NLRP3 inflammasome activation, the production of pro-inflammasome cytokines (such as IL-1B), and ultimately neuroinflammation. Additionally, disruption of LAP/LANDO reduces Aβ species' ability to be phagocytosed and results in neuroinflammation in AD models.

Autophagy modulation is a viable approach for the therapy of neurodegenerative disorders, according to mounting data. The potential of chemical autophagy modulators in the treatment of AD-related pathologies has been the subject of a wide range of studies since 2008, which has increased interest in the development of therapeutics that target autophagy for disease intervention.

Lithium serves as a twofold barrier against tauopathies [29-31]. Tau normally binds to microtubules in transgenic mice to support microtubule assembly, but when tau is hyperphosphorylated, it misfolds, disassembles from microtubules, and forms aberrant filamentous aggregates that result in neurofibrillary tangles. Lithium decreased the activity of GSK3β, decreased tau phosphorylation, and increased tau attachment to microtubules. Lithium reduced mTOR signaling, inhibited GSK3β activity, and ultimately promoted autophagosomal degradation of soluble phosphorylated tau. The autophagic route only digested soluble phosphorylated tau, not insoluble fibrillar tau. Both processes ultimately resulted in improved tau binding to microtubules.

Furthermore, lithium causes a reduction in the level of p62 protein and an increase in LC3-positive puncta in the brain tissue of mice, suggesting that lithium-induced autophagic flux is a key factor in the manifestation of neuroprotective effects [32]. Lithium as a therapy for AD and moderate cognitive impairment has been the subject of numerous clinical investigations. A meta-analysis reveals that lithium therapy may help AD patients with their cognitive impairment, and the results show that lithium administration is safe and tolerable [33-34].

Prion Disease

It is plausible that the fundamental function of autophagy in prion infection has a physiological role and that cells may use it to regulate or combat cellular prion infection [35]. Infectious neurodegenerative disorders known as prion diseases can affect both people and animals. The host-encoded cellular prion protein (PrPc) is thought to create an improperly folded, protease-resistant variant that is the cause of prion disorders [36]. By activating autophagy, 10 mM LiCl decreased the amount of pathogenic prion protein (PrPSc) in murine neuroblastoma cells that were continuously infected with prions [37]. Lithium's anti-prion action was reversed by the treatment of prion-infected cells with 2-methyladenine, a strong autophagy inhibitor, proving that stimulation of autophagy is what causes the destruction of PrPsc.

Lithium was administered to C57BL/6 mice starting 90 days after intracranial inoculation with ME7 prion-infected brain homogenates until their deaths [38]. It was shown that water-in-oil microemulsions with low concentrations of lithium increased the longevity of prion-inoculated mice and reduced vacuolization, astrogliosis, and neuronal loss when compared to controls. These findings suggest that lithium delivered via this novel delivery method may constitute a possible treatment strategy for treating neurodegenerative disorders other than prion diseases.

Amyotrophic Lateral Sclerosis

A disastrous neurological condition called ALS usually results in death three to five years after diagnosis because there is no cure for it. In human ALS patients, daily lithium dosages that resulted in plasma levels between 0.4 and 0.8 mM prevented disease progression [39]. Lithium medication postponed the beginning of the disease and lengthened life span in a study using Cu-Zn superoxide dismutase 1 (SOD1) G93A mutant Cu-Zn male model mice for ALS. In the motor neurons of the spinal cord, the effect was accompanied by an increase in mitochondria, LC3-positive autophagosomes, and autophagy activation. Lithium had an impact on the elimination of protein aggregates and changed mitochondria as well as the synthesis of properly organized mitochondria [40]. Lithium did not have any neuroprotective effects or raise the expression of an autophagy marker in female SODIG93A mice, albeit [41].

The scientists discovered that Li_2_CO_3_ improved tissue viability and reduced the production of reactive oxygen species (ROS) in the developing striatum of rats. Elevated expression levels of LC3-II, LAMP1, Ambra 1, and Beclin-1 were present in conjunction with these beneficial effects. However, Li_2_CO_3_ increased basal oxygen consumption and decreased autophagic flow in the elderly striatum. Aged rats' striatums showed ultrastructural alterations, including lower amounts of normal mitochondria and lysosomes and electro-dense mitochondria with disorganized cristae [42]. As a result, the data indicate that, whereas older rats do not benefit from lithium-mediated neuroprotection, younger animals do. When creating neuroprotective tactics that involve aging-related autophagy induction, these findings should be taken into account.

Given the significant genetic variation in ALS, it appears likely that different genetic subgroups may respond to treatment differently. Using patients with ALS who are homozygous for the C-allele at SNPrs12608932 in UNC13A, a clinical trial (Eudra CT no. 2020-000579-19) intends to demonstrate the effectiveness of lithium carbonate on the time to death or respiratory insufficiency. Fifteen locations across Australia and Europe are going to conduct the study [43].

Parkinson’s Disease

Parkinson's disease is a moderately common nervous system ailment that causes rigidity, gait instability, slowness of movement, and tremors in its victims. There is not currently a neuroprotective candidate that can affect PD disease progression. The hallmark pathological characteristics of PD are Lewy bodies, which are primarily composed of aggregated alpha-synuclein. Using PC12 cell lines, a 10 mM lithium treatment improved the clearance of the alpha-synuclein mutants A53T and A30P by inhibiting IMPase and altering huntingtin [13-14,16]. Rotenon-induced toxicity in human blastoma SH-SY5Y cells, which displayed nuclear fragmentation and apoptosis, was decreased by lithium therapy [44]. With a lithium concentration ranging from 0.2 µM to 10 mM, it was shown that there were more lysosomes and autophagic vacuolar organelles present, as well as decreased reactive oxygen species generation and mitochondrial membrane potential.

GSK3β, the major inhibitor of the WNT/B-catenin pathway, is downregulated by lithium medications. The inhibition of the glutamatergic route, oxidative stress, and inflammation may be related to the stimulation of WNT/B-catenin [45]. Lithium and its numerous and varied interactions with PD may be the subject of upcoming prospective trials.

Ischemic Stroke

In addition to the neurological conditions mentioned above, lithium has also emerged as a possible therapy option for ischemic stroke (IS). Both immediate and delayed neuronal injuries define the intricate pathophysiology of IS. Hypoxia and nutritional deficiency both cause acute cell death in the chronic stage, which sets off processes that cause neuroinflammation and apoptosis. The latter results in a brain injury that is delayed and is further impacted by later vascular reperfusion. Systemic thrombolysis and mechanical thrombectomy are the only treatments available for IS at this time in the acute stage of the disease [46]. In order to slow the evolution of long-term post-stroke syndrome, researchers have been searching for neuroprotective adjuvant treatment strategies. Lithium induces and inhibits certain signaling pathways and effector proteins, resulting in short- and long-term neuroprotection under IS circumstances. As a result, apoptosis, inflammation, and neurogenesis - the three major players in the pathogenesis of IS - are most significantly impacted by lithium (Figure 2) [47].

Neuronal Ceroid Lipofuscinosis

Neuronal ceroid lipofuscinosis (NCL) is a class of severe neurodegenerative lysosomal storage diseases that is among the most prevalent progressive encephalopathies in children [48]. The CLN6 gene has mutations that lead to variant late-infantile NCL. The purpose of the membrane protein CLN6, which is found in the endoplasmic reticulum (ER), is unknown. The pathological indicator of juvenile NCL is the buildup of lysosomes and autophagosomes in central nervous system neurons of a cln3 knock-in mouse model that are rich in the autofluorescence of the mitochondrial ATP synthase complex. Cerebellum development abnormalities and fastigial pathway neuronal loss have been seen in JNCL patients [49]. The restoration of LC3-positive autophagosomes in the cln3 knock-in cerebellar cells was facilitated by 10 mM lithium treatment, and a similar outcome was obtained using an IMPase inhibitor [50]. Additionally, lithium and IMP downregulation prevented cell death brought on by amino acid deficiency.

Multiple Sclerosis

The studies show that low-dose lithium carbonate can be effective in treating progressive forms of MS. However, there is too much variability in the data to draw conclusions about the effects of lithium on brain volume over time [51]. The findings justify the development of a larger, controlled trial with efficacy as the primary endpoint. The initial concern was whether MS patients would tolerate lithium due to its neurologic side effects, such as tremors, fatigue, and cognitive slowing. However, after lowering the daily dose from 600 to 300 mg, tolerance improved significantly, and only one additional subject was discontinued due to lithium intolerance in the following three years. Future studies should focus on the contribution of lithium to symptoms that overlap with MS itself and identify predictive factors for more dramatic examples of intolerance. The serial brain volume measurements suggest a possible stabilizing effect of lithium on brain volume, but differences did not rise to statistical significance [52-53]. Further research should focus on the mechanisms by which brain volume may stabilize or increase.

## Conclusions

In this review, we sought to raise the issue of whether it is justified to treat AD disease with lithium, the prototype mood stabilizer used for both short-term and long-term bipolar disorder treatment. First, we looked at translational research, which highlights the drug's neurobiological properties as shown by its neurotrophic and neuroprotective effects, which are followed by modulation of homeostatic mechanisms like inflammation, mitochondrial function, oxidative stress, autophagy, and apoptosis. We concluded that the inhibition of the IMPase and GSK-3 enzymes by the medication may be the cause of the wide range of intracellular reactions. The reason for the drug's usage in treating AD was further discussed as we went over translational studies in cell culture, animal models of AD and other disorders, and patients. Numerous pieces of evidence from preclinical and clinical investigations support further investigation of lithium's neuroprotective potential in various disorders. This lithium property results from the drug's modulation of several mechanisms and pathways involved in neural plasticity, cell survival, energy-related metabolic activity, transcriptional regulation, and resistance to neurotoxic shocks, as was previously discussed. Some of these mechanisms are fundamentally pathogenic aspects of neurological disorders. Together, the reviewed findings suggest that additional research, including longer treatment durations and larger patient populations, is necessary to definitively confirm or disprove lithium's potential benefits in treating various neuropathies.
